# “Okay, So It’s Not Me”—The *Extra-Fatigue* of Formal and Informal Reporting of Sexual Harassment in Academia

**DOI:** 10.3390/ijerph23050634

**Published:** 2026-05-11

**Authors:** Oriana Binik, Debora Ginocchio, Clarissa Cricenti, Silvia Ferrari, Tindara Addabbo, Isabella Merzagora, Anna Maria Giannini, Georgia Zara, Giovanna Laura De Fazio

**Affiliations:** 1 Department of Law, University of Milan-Bicocca, 20126 Milan, Italy; oriana.binik@unimib.it; 2 Department of Law, University of Modena and Reggio-Emilia, 41121 Modena, Italy; giovannalaura.defazio@unimore.it; 3 Department of Psychology, University of Rome “Sapienza”, 00185 Rome, Italy; clarissa.cricenti@uniroma1.it (C.C.); annamaria.giannini@uniroma1.it (A.M.G.); 4 Department of Biomedical, Metabolic, and Neuroscience Sciences, University of Modena and Reggio-Emilia, 41122 Modena, Italy; silvia.ferrari@unimore.it; 5 Department of Economics “Marco Biagi”, University of Modena and Reggio-Emilia, 41121 Modena, Italy; tindara.addabbo@unimore.it; 6 Department of Biomedical Sciences for Health, University of Milan, 20133 Milan, Italy; isabella.merzagora@unimi.it; 7 Department of Law, University of Turin, 10153 Turin, Italy; georgia.zara@unito.it

**Keywords:** sexual harassment, academia, reporting, extra-fatigue, prevention, narrative criminology

## Abstract

**Highlights:**

**Public health relevance—How does this work relate to a public health issue?**

- Examines how victims of sexual harassment in academia interpret their experiences and navigate informal and formal reporting pathways within a socio-ecological framework.
- Explores how relational, organizational, and cultural factors shape coping strategies and access to support, influencing health and well-being outcomes.

**Public health significance—Why is this work of significance to public health?**

- Reveals a critical mismatch between supportive informal networks and inadequate or dismissive institutional responses, limiting effective help-seeking and reporting.
- Highlights how ambiguity, lack of validation, and fear of retaliation act as structural barriers, exacerbating psychological distress and reinforcing underreporting of sexual harassment.

**Public health implications—What are the key implications or messages for practitioners, policy makers and/or researchers in public health?**

- Calls for trauma-informed, transparent, and victim-centered institutional procedures that align with and strengthen existing informal support networks.
- Supports the development of community-based and bystander-informed interventions to improve recognition, response, and prevention of sexual harassment in academic settings.

**Abstract:**

Sexual harassment (SH) in academia constitutes a pervasive form of gender-based violence that undermines individual well-being and academic equity. While existing research has largely investigated risk factors, less attention has been paid to protective factors, coping strategies, and the cumulative burden generated by the reporting process itself. This qualitative study explores how victims of SH in Italian universities mobilize resources when disclosing their experiences informally to peers and colleagues or formally through institutional channels and how this process generates *extra-fatigue*: the cumulative cognitive, emotional, and practical labor victims have to perform as a direct consequence of inadequate institutional responses. Drawing on semi-structured interviews, we employed thematic and dialogical narrative analyses to examine cognitive, emotional, and behavioral coping dimensions. Findings highlight the central role of informal networks in enabling victims to recognize harassment, validate their narratives, and mobilize coping strategies. Trusted colleagues and supportive professors provided cognitive clarity, emotional relief, and practical protection. However, institutional responses were frequently perceived as inadequate or emotionally detached, reinforcing self-doubt and generating significant extra-fatigue to absorb largely alone or through informal support. Understanding extra-fatigue as structurally produced labor, rather than individual fragility, has implications for designing victim-centered institutional responses and structural reform in universities.

## 1. Introduction

Sexual harassment (SH) is a form of gender-based violence (GBV) that encompasses «any unwelcome sexual advance, unwelcome request for sexual favor, verbal or physical conduct or gesture of a sexual nature, or any other behavior of a sexual nature that might be expected or be perceived to cause offence or humiliation to another» [1]; as such, it can create an intimidating and hostile environment. It affects individuals across several settings, including academia, where it not only undermines personal well-being but also impedes equal access to education and professional opportunities [2,3]. Thus, addressing SH is essential to promoting respectful and equitable academic environments.

Over the past decade, attention to SH has grown, beginning on U.S. campuses [4] and later expanding globally. Many recent investigations [5,6,7] have emphasized the widespread nature of the problem, shedding light on its patterns, frequency, and the serious consequences. Indeed, several programs and policy efforts across Europe have emerged to prevent GBV and SH in academic settings [8,9,10,11,12]. Regarding vulnerability, a recent systematic review [7] confirmed that macro-dynamics of power, social inequalities, and cultural marginalization are risk factors for SH in academia: women, LGBTQ+ individuals, people of different ethnicities, or indigenous populations are most at risk [13,14,15,16]. Previous experiences of discrimination also increase risk, as do asymmetrical relationships: students are particularly at risk of victimization by administrative and academic staff, as are young academics by those in higher positions [14,17,18,19].

Since much of the research on SH in academia has focused on risk factors, overlooking the crucial role of protective factors [20,21,22], it is important to integrate this field of study with that of coping strategies, as they are closely related. To understand how protective factors and coping strategies operate, it is essential to look beyond the individual and consider the broader socio-cultural and organizational contexts that shape victims’ experiences and decisions [23,24]. This is especially relevant for SH, where ambiguity often hinders victims’ ability to recognize and interpret the situation [25]. Many survivors prefer to speak to informal contacts rather than formal services; thus, empathic availability of peers and colleagues becomes crucial, as supportive responses can encourage further help-seeking [26,27]. However, little research has explored these dynamics in academic settings.

Based on this, after reviewing the literature on these topics in the following sections, this qualitative study uses a narrative approach to examine how victims of SH in academia make sense of their experiences and what coping resources they mobilize when choosing to speak out. By analyzing both informal and formal reporting, the research shed light on the interplay between individual, relational, and contextual levels in the seeking-help process.

### 1.1. Protective Factors and Coping Strategies

Most researchers concur that protective factors either lower the probability of a negative outcome occurring or help mitigate existing risk factors by functioning as mediators or moderators (buffering protective factors) [28,29]. Focusing on protective factors for victimization is important to strengthen resources of potential victims, buffer SH effects, and reduce the harm. Some studies identified assertiveness as a key characteristic in coping with such risky situations: women who respond assertively, by actions like running away or yelling, are more likely to prevent a sexual assault than those who react passively, such as freezing or crying [30,31,32]. An important factor in assertive responses is efficacy to resist or confidence to act against a potential perpetrator [33,34,35], which may help women also deter SH, potentially reducing their risk of being targeted [36,37]. At an interpersonal level, Perez-Trujillo et al. [38] argued that connectedness, i.e., feeling included, respected, and supported within social groups and institutions, can serve as a protective factor by limiting exposure to potential perpetrators and enhancing peer support in risky situations, both of which lower the risk of victimization. As for organizational level, committed leadership, strict no-tolerance policies, and frequent evaluation of the organizational climate are commonly associated with lower SH prevalence [39,40].

Closely related to protective factors for SH victimization are coping strategies to manage the experience so as to minimize its impact. There has been growing scholarly interest in how women navigate SH incidents, highlighting the variety of strategies used to deal with such situations [23,41,42,43,44]. These range from passive approaches—e.g., ignoring the perpetrator, downplaying, or blaming themselves—to assertively active ones, like seeking help through formal reporting or informal disclosure to significant others (friends or family) or confronting the harasser [45]. Fitzgerald [46] developed a framework based on data collected from victims of workplace SH, originally drawn from a large-scale prevalence study [47], which outlined several SH coping strategies divided into two broad categories: internally and externally focused responses, comparable to the previous, more general distinction between emotion-focused and problem-focused coping strategies [48,49], Internally focused strategies aim at managing thoughts and emotions, such as acting as if the behavior has no impact (denial) or finding alternative explanations or rationalizing the perpetrator’s behavior (reattribution). On the other hand, externally focused strategies are action-oriented and address the situation, including distancing oneself from the harasser, using humor, excuses, or delay tactics to deflect further behavior, or seeking help. Formal responses, like filing complaints or taking legal action, are relatively rare and often come after all other efforts have been exhausted [41,43]. Avoidance is one of the most common external responses, while humor is a frequent appeasement tactic; seeking support from friends or colleagues is also widespread [23,45,50,51,52].

Recently, research on protective factors and coping has become important for prevention. Although the path is long and presents several critical issues, it is now possible to raise awareness of commonly used coping strategies within academia and develop training to practice life skills and protective factors involved, such as assertiveness, effective communication, and emotional management [20].

### 1.2. Relational and Community Support

To grasp how protective factors and coping strategies work, it is necessary to move beyond focusing solely on the person and consider the broader context, including socio-cultural and organizational factors, that shape victims’ experiences and choices [23,24].

The social-ecological framework (SEM) [53,54] emphasizes the supportive role of relational and community context: preventing SH requires not only strengthening the internal resources of the individual, but creating a virtuous cycle of mutual reinforcement of individual, relational and contextual protective factors that has been pioneered in health promotion over the past 40 years [55] and recently adopted in the field of sexual violence prevention [56]. The desired outcome is an entire academic community with the necessary resources to identify and cope with SH instances. If someone becomes a victim, the SEM is committed to ensuring that they have access to support within their close-knit relational network, as well as within the institutional tertiary prevention services [57].

This is crucial in the case of the “gray area” of SH, which may create a strong sense of ambiguity and difficulty in understanding the situation [58]; by way of example, recent data from the UniSAFE project on GBV and SH in European research-performing organizations (https://unisafe-gbv.eu/) show that 31% of the more than 42,000 total respondents did not recognize the behavior as violence at the time of the incident [59]. Underlying this feeling of uncertainty is both the concept of «hermeneutical injustice» [60,61], referring to the fact that targets are unable to understand what is happening to them «owing to a structural identity prejudice in the collective hermeneutic resource» [57] (p. 155), and that of «gaslighting» [62,63], when victims are deliberately deceived and manipulated about what is happening to them, leading to what Fernando & Prasad [64] have defined as «reluctant acquiescence». In this context, social support can play a key role in helping victims to understand what is happening, process their experiences, and finally move forward [65]. However, breaking the silence and allowing the relational and contextual levels to support could be a critical step [66]: victims often choose not to report because they consider the episode insignificant [23]; for fear of retaliation or of being blamed [67]; for the shame associated with the incident, or a lack of confidence in effective complaint handling [68]. One way to overcome these barriers is to make relational and community contexts sensitive and ready to support victims in coping. Consistently, previous studies on survivors of sexual assault show that most individuals choose to share their experiences with informal contacts, rather than turning to official support services. The nature of the responses from these informal sources plays a crucial role in shaping survivors’ willingness to seek further help, with supportive and validating reactions increasing this likelihood [26,27].

Currently, there is limited literature on these specific aspects of SH in academia. A study by Vargas et al. [69] on identity-based harassment found that individuals who chose to report in the academic context encountered institutional minimization or retaliation. These findings are consistent with research in other professional contexts and concerning other types of harassing behaviors, which has shown that speaking out about mistreatment may result in demotion, professional marginalization, and social isolation, especially when the perpetrator holds a position of power [70]. Also outside the professional context, Pijlman et al. [71] found that SH victims help-seekers reported greater experienced barriers to help-seeking, when compared to non-help-seekers.

Within this framework, Kirkner et al. [72] emphasized that there is little research focusing on seeking help and reporting SH in academia and called on future scholars to adopt qualitative approaches to better understand the complex dynamics of reporting in academic workplaces. Understanding SH reporting in universities requires attending to the cultural and structural conditions that shape it. In the Italian academic context, these include the intense dependency between junior academics and individual supervisors, the widespread use of temporary contracts that suppress formal reporting, and the non-binding advisory status of the *Comitati Unici di Garanzia* (CUGs, the primary institutional mechanism for equality and well-being in Italian public universities)—conditions that actively shape both the production of harassment and the conditions under which victims can or cannot report it [73,74,75]. Ahmed [73] documents how institutional complaint procedures frequently operate as non-performatives: they circulate as if they matter—as if they address the problem—while leaving intact the very power dynamics that make complaints necessary in the first place (p. 52). Understanding reporting failures in Italian academia therefore requires attending not only to individual responses but to the structural conditions that make formal procedures systematically inadequate.

In light of this, it would be desirable to deepen these aspects for prevention purposes because it would allow protective factors and coping strategies to be placed more precisely within the broader relational and community framework. This would enable strategies to be identified that would make the academic community aware of the importance of informal support and tertiary prevention dedicated services.

## 2. Materials and Methods

Beyond the limited knowledge about seeking help and reporting SH in academia, the research methods are mainly quantitative, which, although certainly useful and necessary, fail to capture equally crucial aspects, such as the experiences and interpretations of those involved.

Narrative approaches to victimization experiences should enrich the field of prevention in many directions. As argued by Pemberton et al. [76], narrative victimology has an emancipatory potential, because it focuses on victims as individuals who have and make choices (e.g., to seek help or not); then, attention is on coping mechanisms not in abstract, but as strictly connected to victims’ interpretations of the situation. Finally, narrative approaches seek to query the interaction of the victim’s story with other stories related to the event, such as the colleagues’ narratives and the services’ narratives. In sum, narrative approaches can be useful in reconstructing stories of victimization that are not sterile but enriched by the subjectivity of the people involved and how their stories intertwine [76,77]. The narrative turns in victimology allows for more in-depth and nuanced insights into victims’ experiences, that are often simplified or even excluded in the representations of victimhood found in crime statistics [78].

Based on this, this qualitative research aims to investigate resources and coping strategies that can be activated when someone experiences SH and decides to step out of the shadows, turning to the closest colleagues and superiors, or initiating the formal reporting procedure. Through a narrative approach, the focus is not on the events themselves, rather on how they are interpreted by the victims and how their stories are co-constructed in interaction with others [76,79]. Indeed, personal narratives take shape and gain meaning through their continuity with the stories of others, all of which are influenced by the broader cultural narratives [80,81]. This is crucial for victims, as experiences of victimization often disrupt the link between their personal narrative and the surrounding context, thus making them feel doubtful about their viewpoint [82]. This is particularly true in the case of SH victims, who need to overcome ambiguity and difficulties in understanding the situation [83]. Rebuilding that connection through social support and the validation of their stories can play a key role in helping victims cope and process their experiences [58,65].

### 2.1. Objectives

The focus of this qualitative study is on victims’ narrative redefinition through contact with others, i.e., in seeking informal or formal help, and how this influences the coping dynamics. In the logic of the SEM [53,54], this means reconstructing the red thread that exists between the individual, relational, and contextual levels. This allows for the (re)design of effective dedicated services that truly take the victim into consideration, avoiding secondary victimization and barriers along the help seeking and reporting processes.

Specifically, through a semi-structured interview with victims of SH in Italian universities, the following objectives will be pursued:

1. To shed light on resources and coping strategies mobilized by victims to deal with the situation, focusing on how individual narratives about the help seeking path intertwine with relational (network of people), and contextual (services offered) narratives.
2. When the victims decide to formally or informally report within university, to understand the impact perceived by victims in reorienting their narratives, resources, and ability to cope with the situation, both internally—managing personal emotions and cognitions—and externally (action-oriented responses, implemented at the behavioral level) [46].

Investigating the light and shadow of the SH coping and reporting processes can provide useful insights to develop effective and victim-centered interventions and services, to empower the academic community, prevent secondary victimization, and minimize the consequences of SH in academia.

### 2.2. Sample and Procedure

We developed a semi-structured interview protocol to collect data from narratives of victims of SH in Italian universities. An initial open-ended question aimed at gathering the victim’s experience(s) in narrative form, thus keeping track of the story and its developments, was followed by questions exploring the dynamics of coping with the situation. Specifically, the script consisted of three sections following the initial open-ended question asking participants to reconstruct the experience:

1. Coping strategies (cognitive, emotional, and operational/behavioral) at an individual level to manage the situation (e.g., in response to what happened to you, how did you react/what did you feel?)
2. The relational context, whether supportive or not, with respect to the incident (e.g., was anyone else present during the incident or was anyone else aware of it? (If yes) Did they do anything?)
3. Resources at the institutional level (e.g., were there services at the university that you considered turning to? (If yes) What was your experience in this regard?).

Several channels were used to disseminate the study, including: 1. notices and posts on university communication channels and social media; 2. activation of the CUGs network so that the research reaches as many Italian universities as possible; 3. posting flyers in university spaces; and 4. posts by activist and feminist social media profiles with a considerable number of followers.

To get in touch with potential participants, an institutional email address has been created to which emails expressing interest in taking part in the study should be sent. The response to the initial contact from the candidate described the study, providing all necessary information, including the guarantee of anonymity and the requirement to sign an informed consent form. Interview appointments were then scheduled with those who agreed to participate.

Between January and June 2025, *n* = 12 interviews, lasting an average of one hour, were conducted online by two researchers; however, in *n* = 4 cases the victims did not seek support from anyone within the university, either formally or informally. Therefore, the data coding was limited to those *n* = 8 interviews in which formal or informal reporting took place. The descriptive details of the interviewees are shown in Table 1, where one case departing from the dominant pattern should be noted: a male professor targeted by a female student, representing a contra-power configuration in which institutional seniority and professional authority ran counter to the direction of the harassment. Analytically, this case is instructive precisely because many dynamics identified across the other narratives and discussed below were substantially reproduced. This suggests that the mechanisms described in this paper are not reducible to gendered vulnerability alone, even as gender structures most cases. The intersectional character of power—operating through gender, seniority, institutional role, and relational context simultaneously—shapes both the experience of harassment and the conditions of its reporting, as the existing literature on contra-power harassment confirms [84].

Consistent with the epistemological premises of narrative victimology, the adequacy of the sample is assessed not in terms of statistical representativeness but in terms of the depth and diversity of the accounts collected [81,85]. Participants were included in the analytic sample if they had experienced SH in an Italian university, had engaged in at least one form of informal or formal reporting within the university, and were willing to provide informed consent; the four participants who did not report to anyone within the university were therefore excluded from thematic coding, though their accounts were read and discussed as analytic counterpoints (see Section 5). In narrative inquiry, smaller samples are appropriate—and indeed preferable—when the aim is to examine how individuals make sense of their experiences, reconstruct meaning through interaction with others, and navigate institutional processes [86]. Eight narratives involving formal or informal reporting allowed for theoretically meaningful comparison across academic roles, types of reporting, and institutional responses, while preserving the richness and contextual specificity that characterize this approach. Thematic saturation across the three core dimensions—cognitive, emotional, and behavioral—was reached by the fifth interview; at that point, subsequent accounts were confirming and deepening existing themes without introducing substantively new ones. Rather than closing recruitment at that point, however, we deliberately extended data collection to seek greater variation in participants’ accounts of their institutional experiences, diversifying dissemination channels to reach individuals who might report different types of formal or informal responses. The three additional interviews collected through this extended recruitment did not produce new themes but confirmed the robustness of the emerging framework across different institutional contexts and reporting trajectories. This combination of thematic saturation and active search for variation is consistent with theoretical saturation in narrative inquiry [87] and reflects a deliberate methodological choice to strengthen the credibility of the findings rather than to close recruitment at the earliest point of saturation.

At the same time, the sampling strategy introduces a selection bias that warrants explicit acknowledgment. Participants self-selected in response to institutional and activist channels, and all but one identified as cisgender women. This reflects a well-documented pattern in research on gender-based violence, whereby those who respond to calls for participation are typically individuals who have already achieved some degree of narrative coherence around their experience and feel sufficiently safe to disclose it [66,67]. Marginalized groups—including LGBTQ+ individuals, people with precarious immigration status, and those facing intersecting forms of discrimination—are likely underrepresented, as they may perceive greater risks in coming forward. Findings should therefore be read as accounts of those who were able and willing to speak, rather than as representative of the full range of SH experiences in Italian academia. This limitation underscores the need for future research that employs targeted recruitment strategies to reach more marginalized populations.

Starting with an open-ended question fostered natural storytelling, letting participants express what mattered most in their own words, with minimal prompting and interruptions [85]. Considering that victims are often required to tell their stories within rigid institutional settings, like courts or police interviews, a participant-led method is valuable in narrative victimology, as it enables richer, more authentic insights into their experiences [78].

### 2.3. Data Analysis

The data from the interviews were analyzed independently by two researchers in relation to two main levels [86]:

1. Thematic analysis of the narratives about responses and resources activated by the victim internally (management of cognitions and emotions) and externally (behavioral or problem-focused level [46,48,49].
2. Dialogical analysis of the multiple voices included in victims’ narratives, with a focus on formal or informal reporting processes within academia.

The data analysis focused on how these two levels intertwined, exploring how informal (relational level) and formal (contextual level) reporting influenced the individual narratives, i.e., the victim’s internal (emotions and cognitions) and external (behavior) responses [46]. In other words, a particular emphasis has been placed on the analysis of the consequences that these stories have had on the victims who have chosen to seek help and, consequently, to assume the role of storyteller.

The two researchers conducted independent coding passes across all 8 transcripts before convening to compare analyses. Initial discrepancies arose primarily at the sub-theme level—for example, in distinguishing cognitive intellectualization from behavioral problem-solving in passages where participants described research-driven sense-making—and were resolved through iterative discussion until full consensus was reached. All coding decisions and revisions were documented in a shared audit trail, providing a record of analytic reasoning throughout the process. In line with consensual qualitative research conventions [88], no quantitative inter-rater reliability coefficient was calculated; instead, analytical rigor was ensured through the combination of independent initial coding and documented consensus procedures. The process of identifying themes and subthemes and coding them is described in the Table S1.

The dialogical dimension of the analysis attended specifically to shifts in voice within individual narratives: moments where participants moved between self-doubt and self-assertion, between the minimizing language absorbed from institutional encounters and the validating language recovered through informal relationships. Operationally, these shifts were identified through markers including pronoun instability (“I thought it was my fault… but then she said…”), reported speech in which others’ words visibly reorient the narrator’s self-assessment, and evaluative contrasts within a single turn of speech. In the Results section, each excerpt is introduced with a brief framing that makes this dialogical layer explicit, drawing the reader’s attention to the specific voice dynamics at work in the passage.

Given that not all respondents’ universities had dedicated services, when SH occurred the two coders decided to consider formal reports also when the victim contacted the Rector or Department Director, and informal reports when the person disclosed the incident to colleagues in an equal position or to trusted superiors. It must be acknowledged that this classification involves a degree of interpretive judgment rather than a strict procedural boundary: in the Italian academic system, the formal complaint procedure formally requires the involvement of the Confidential Counselor—an external lawyer to whom employees and students can turn for advice and help in cases of violence, harassment, or discrimination—yet several cases in this sample predate the widespread institutionalization of such procedures, making a strictly procedural classification anachronistic. Rectors and Department Directors have therefore been classified as formal interlocutors on functional rather than procedural grounds, as institutional authorities whose responses carry organizational weight, accountability implications, and the capacity to produce institutional consequences that peer responses structurally cannot. This functional criterion is consistent with a reading of formality as a continuum rather than a binary.

### 2.4. Researcher Positionality

Both researchers conducting the interviews are affiliated with Italian universities and, therefore, are embedded in the same structural and cultural context they are analyzing, including its hierarchical dynamics, precarity conditions, and gendered power relations. Notably, one researcher had navigated a prolonged period of academic precarity prior to this study, while the other was at an early stage of her academic career at the time of data collection; the experience of institutional precarity was therefore not merely an analytical category but a lived dimension of the research relationship, and its implications for interpretation—including the risk of over-identification with participants’ accounts—were explicitly discussed during the analysis phase. This positionality was managed through ongoing reflective practice: interviews were deliberately scheduled to allow adequate recovery time before subsequent professional commitments, recognizing the emotional weight of the material. Researchers maintained individual reflexive notes throughout the data collection and analysis phases and engaged in regular debriefing conversations with each other and with one of the study supervisors to identify and bracket assumptions that might shape interpretation. Interviews were emotionally demanding, and researchers made use of peer support and, where needed, supervision to process vicarious exposure to accounts of harassment and institutional failure.

## 3. Results

Data from these interviews were categorized into three main themes of narratives: 1. Cognitive dimension, 2. Emotional dimension, and 3. Behavioral (problem-focused) dimension. These, in turn, have been coded and divided into the following topics.

### 3.1. Narrative’s Cognitive Dimension

#### 3.1.1. Framing the Situation Through the Eyes of the Others

Given the ambiguous nature of SH, the first step that victims often face is framing the situation they have experienced. Since such incidents are not always explicit, the victim may question whether they have misunderstood the situation. Sexual comments or advances, although unwanted, fall into that insidious gray area that is less easy to define as inappropriate, even in the eyes of the victims, even though they feel uncomfortable and harmed. Talking about it with others can help frame the situation, understand that it is SH, and that “*it’s not me*”. The external gaze is necessary to make one’s own narrative more coherent, thus allowing one to direct action: external validation of personal narrative is therefore a crucial condition for beginning to cope with the situation directly. This can happen in a formal reporting, as in the case of one interviewee, whose words clearly convey this concept: “*I decided to go and talk to the Confidential Counselor, who told me ‘What you experienced can be defined as SH’. So, I told him (the perpetrator) straight out ‘What you’re doing is harassment’. I also said ‘You’re lucky you did it to me, that I didn’t know, that I’m also fond of you, I’ll let it go, but you have to stop because it’s not right, I can’t accept comments like that from you anymore’*”.

But awareness of the situation can also arise in informal help seeking, as happened to some interviewees who turned to colleagues or expert and authoritative figures: “*It was a bit informal. I talked about it with a friend and colleague of mine […], she helped me a lot because she gave me a wake-up call and said ‘I think this is a form of harassment. I’m no expert, but I think so’. She referred me to another professor […]. This professor was very harsh, but in a good way: she shook me up and said ‘This is harassment, you need to contact this service’*”.

However, it can also happen that the external context does not validate personal narratives, causing self-doubt. In this case, co-narration tends to be the result of internalizing the interpretations of others, leading to inner discomfort. The next excerpt demonstrates the opposite dialogical movement: rather than validating her narrative, the surrounding context introduces a minimizing voice that the narrator progressively internalizes. The shift from external dismissal to self-doubt—*“I realize that I have internalized this a lot*”—exemplifies the dialogical collapse that follows the absence of validation: “*The context around me told me that it was no big deal, that this person was perhaps misunderstood, he probably needed to learn to manage his interactions with students better, because maybe he’s a bit inappropriate. But no clear stance was taken to protect me, like ‘It’s serious that he did this’, but rather ‘What a pity that there was this misunderstanding’. I realize that I have internalized this a lot*”.

Another way to frame the situation with others may be through peer support. Those who have had the same SH experience can act as a mirror and help to put a name to it. In addition to cognitive clarification, seeking comparison with others offers the possibility of escaping from a condition of loneliness and self-blame. In essence, if it has happened to other people too, there is a real problem, and this problem is not the victim’s misperception. The following excerpt illustrates a pivotal dialogical shift: the narrator moves from an internalized minimizing voice—questioning her own perception—to a validated self-assertion, made possible only through the mirroring account of a peer. Note how the shared experience does not merely provide information but reorganizes the narrator’s self-assessment: “*I contacted a colleague of mine and she was receiving the same advances. We began discussing it and trying to figure out how to handle the situation. As bad as it was, though, having someone who was going through the same thing at that moment helped me, and I thought ‘Okay, so it’s not me’”*.

In some cases, this peer support was such an empowering resource that it generated thrust and confidence to proceed with a formal report: “*I got in touch with a second person, who told me about her personal experience ‘He contacted me in exactly this way […], then it turned into a two-year nightmare’ […]. She told me her perception of this man […], we discovered that he had this pattern with various people […]. Anyway, she and I decided to report it, going to the Confidential Counselor*”.

#### 3.1.2. “If I Don’t Understand, I Try to Intellectualize It”

Consistent with the academic context, studying has sometimes helped victims make sense of their experiences, finding the words to frame them: some interviewees threw themselves into their studies to conceptualize, understand, and thus be able to move forward. The use of this rationalization mechanism occurred either at the suggestion of someone already interested in these issues, or as a personal drive to fill the inner void due to the lack of support from the network around them. These are two conditions at opposite ends of the relational continuum, in which either the network constitutes a resource, or it does not exist and is not supportive, and thus study becomes a refuge from this loneliness. The following excerpts are illustrative examples.

“*I was talking to another professor […] about harassment in general, about problems at university; she said to me ‘You should read Simon de Beauvoir. You girls don’t read anymore, but you should’. That’s when I started reading lots of feminist literature of various kinds […]. Exploring this topic, always from an intersectional feminist perspective, has opened my mind. I started to reinterpret my own experience in a different light, I became aware […]. That was the moment when I said ‘Now I have to do something. I won’t change the world, but this is intolerable’. It was a gradual process […], pieces that came together that made me aware*”.

“*There was this intense isolation that made me feel very lonely. There was this thing of seeking justice for myself, which caused me a crisis of confidence:‘ Am I really right? Am I misunderstanding everything?’. I still haven’t resolved this […]. This thing affected me a lot, indeed after this event, I’ve started studying this topic […]: the literature agrees with me and I can also say that it’s true*”.

Named the situation interacting with others, if this sharing has taken place informally, this initial step can lead to formal reporting. In this case, narratives from interviews show additional difficulties in the reporting process: even if it is recognized that this is SH, the victims do not feel the help they need and the recognition and validation at the institutional level. The victim is once again faced with an inconsistent and ambiguous situation that is difficult to accept. Therefore, additional resources must be activated, another cognitive “extra-fatigue” is caused by the need to make sense of the system’s immobility or unsatisfactory responses. In some cases, intellectualization and reliance on scientific narratives are also used to explain the immobility of dedicated institutions. Explanations on “*internalized misogyny*”—quoting the words of an interviewee—and a patronizing system appeared, which were reached both through personal reflections and, once again, through scientific study. The idea is that many people have long embraced values that make them think certain inappropriate behaviors are normal; therefore, the only solution is simply to accept them. Below is a significant excerpt: “*I was really disappointed by university […]. I never expected anything like that and I’ve been thinking about how to explain it. I’ve been thinking about various things I’ve read in other fields, but always on similar topics, so I’ll try to conceptualize it. There are many people, including many women, who probably grew up in an era where there were many changes, many revolutions, but not all of them experienced them firsthand. Many people lived in contexts that were still patriarchal, rigid, much more closed to external stimuli and, therefore, internalized this vision*”.

### 3.2. Narrative’s Emotional Dimension

#### 3.2.1. Openness and Emotional Support from the Academic Network

Interaction with others not only helps in understanding what is happening but is also essential for managing negative emotions associated with the SH incident. The role of the bystander is crucial on an emotional level as well: as in the cognitive case, a bystander is not only someone who directly witnesses harassment, but also those who have the sensitivity to embrace the resulting emotional distress, supporting the victim in managing the suffering. In the following excerpts the informal intervention by professors supports victims in recognizing their value, taking away the power of antagonistic narratives aimed at arousing shame and self-blame. Moments of emotional co-management emerge and the more sensitive side of the academic world appears: the relational proximity typical of this context is protective, suggesting the presence of deep relationships based on the ability to see and accept the value and emotional experiences of younger people.

“*For the umpteenth time, I arrived at university in tears; my current supervisor welcomed me, because after yet another phone call in which I was told ‘I’ll tell everyone who you are’, he welcomed me and said ‘We all know who you are, don’t worry, no one will believe the word of someone like that’ […]. From there, I have to say that it actually died down. I didn’t make any formal communication or report, beyond informing my colleagues and the department director, but this had no follow-up or outcome. The only person who made me feel a little reassured was my supervisor*”.

“*The only person who did that was my supervisor […]. Once, it was late, I couldn’t sleep because I felt terrible, I called her in tears, we talked on the phone for an hour: she gave me enormous support, telling me that ‘The space belongs to you, they took it away from you, you’re winning it back; if someone looks at you badly, look at them worse, because you have nothing to be ashamed of’*”.

In both these cases, the ability to listen and the weakening of blaming narratives play a crucial role in the events, prompting victims to take action to stop SH. However, not all bystanders may have the ability to support the emotional experience of victims in such a profound way. Other situations suggest the presence of a network of less deep connections in the academic context, which, however, play an equally important role in helping victims. Such support can take the form of a passing conversation, in which a teacher suggests that anger is the most appropriate emotional response to SH, or a secretary offers a moment of decompression in her office to a professor who has just been kissed against her will by one of her superiors.

“*I was also working with another professor and I told him about this because I trusted him, and he got really angry. He said, ‘This is unacceptable’. Maybe it’s because he’s foreign and he has a different mentality and approach*”.

“*There was hardly anyone left […] I went to the office of the department secretary, who was staying late, and I told her what had happened. I asked her if I could stay there for a while, because I didn’t want to go out and risk running into him, feeling like that. She was an elderly lady, very maternal, who understood. So, I stayed there and I remember at that moment realizing that a line had been crossed*”.

#### 3.2.2. The Emotional Extra-Fatigue: Dealing with Distress and Anger

Exposing vulnerability and sharing one’s story in a formal context is often the result of a bumpy path, which requires the mobilization of many resources to arrive at the hope that reporting will bring justice, putting an end to SH. Informal support from peers or welcoming teachers is often a prerequisite for initiating a formal procedure and bringing the matter to a conclusion in which one’s narrative is recognized at an institutional level, raising a sense of peace. Once they reach this level of awareness, if victims encounter belittling narratives, lack of empathy, or system inefficiencies, there may be a setback in the process of awareness and coping, unexpectedly intensifying feelings of distress, fear, and loneliness. The first of the two sample excerpts below captures a dialogical rupture between the victim’s expectation of institutional recognition and the bureaucratic voice she actually encountered. The narrator enters the formal process carrying the validating narrative built through informal support; the institutional response introduces a coldly procedural counter-voice that displaces that narrative, producing the emotional isolation described.

“*I went to the Confidential Counselor, it wasn’t a very positive experience because I found her to be a person with zero empathy, she was very cold. She asked questions… OK, she was doing her job, which I don’t think is to console people, but she was very cold, like a bureaucrat to whom I was telling my details to get my ID card. Then she said to me ‘Well, if you don’t have any recordings, any evidence, there’s nothing you can do, I’m sorry, because according to Italian law…’. She explained the law to me. I don’t remember anything, because I was in a trance, I couldn’t even understand what she was saying. I said to her, ‘The moral of the story is that there’s nothing I can do’. ‘No…you should try applying abroad, because these things happen less’. It was a truly useless and even somewhat negative experience, because it made me feel very alone*”.

“*I felt very exposed and alone and this is still a topic of therapy, because, deep down, I also think ‘Come on, they were just messages, you’re making a big deal out of it’. […] Being afraid of running into someone when you’re in the hallway, which is awful anyway; then having to assert that I’m right because they’re not agreeing with me here and not protecting me, indeed they suspect that this person may just be being nice and that I’ve misunderstood him. I really feel that this weighed heavily on me emotionally*”.

This last excerpt suggests that the feeling of having brought a narrative into the public domain, that perhaps should have remained private, can return victims to a feeling of ambiguity, beginning to doubt the “truth” of their own story. Once again, the narratives surrounding the event become contradictory: while some close people had given coherence to personal narrative, providing words and emotional support to understand what had happened, others, in a formal setting, may act differently. As a result, the belittling narrative in the institutional setting can cause embarrassment for having brought intimate issues into a context that sometimes seems to lack the resources to truly take care of these experiences.

“*I went to talk to the department director, because I started thinking, ‘Well, I have to protect myself, because if he goes around saying that I’m a certain type of person, that I’m taking advantage of him, I have to protect myself and prevent it’. He said to me ‘Yes, I know he’s a peculiar person,’ but he made it clear to me that the matter should end there. […] He had belittled it a little […] I remember that I left there feeling embarrassed*”.

In some cases, loneliness and embarrassment can give way to anger, a more active emotional experience. The emotional turning point occurs when certain pieces are added to the narrative, revealing the presence of other victims, or when the informal context or the information gathered confirms the consistency of one’s story, challenging system inefficiencies.

“*After a year, it emerged that this person had a […] disorder that he didn’t disclose […]. At that point, the person who was running the center where I work asked me to file a complaint, and I said no, because I was understandably angry: when it suited the university, my testimony was useful, while for the entire first year in which I reported harassing incidents, I was taken lightly. […] Especially during the first year, when I reported these incidents and was not believed, it was quite distressing*”.

“*I started reading on the phenomenon, books by feminist authors. I became increasingly aware of this issue and then I felt really angry towards him, towards my family. I’m sorry to say this, but it’s the truth. I felt a lot of anger towards the people in that university, because obviously I then started talking about it with some trusted professors […] they really belittled me, and I’m sorry to say this […]. One professor said to me ‘Come on, it’s just a joke, you girls today are too uptight’. It doesn’t seem like just a joke to me*”.

Managing this emotional tangle requires emotional extra-fatigue, added to that generated by SH. In some cases, psychological support from university or private services was necessary for emotional management. In others, faced with a lack of response because the situation was very complicated, the fear and anxiety associated with persecutory behavior, which could not be stopped even after the report, was managed through the solidarity of the group of complainants.

“*The Confidential Counselor arranged five sessions with the psychologist of the university, who was very good, a nice woman […] the only truly positive figure. She was good, but it’s probably also a personal thing. She was the only one who was truly shocked, who made me talk*”.

“*We were all scared, because if it had happened to someone else to be touched, who could say that it couldn’t happen to us too, given that we were in the same situation? […] It was a closeness, that’s what it was […] with an event like that, we became close friends […]. During that particular period, we often slept together […]. This helped us a lot too, because what we all felt at the beginning was helplessness and the fact that we had all fallen for the same excuse*”.

### 3.3. Narrative’s Behavioral Dimension

#### 3.3.1. Operational Coping Strategies

As discussed so far, dealing with SH requires cognitive and emotional resources, as well as the ability to implement behaviors that curb it. Once again, the focus is on the relational dimension, i.e., how the intertwining of narratives leads to the implementation of behavioral strategies aimed at protecting victims.

Sharing an operational strategy of behavior is found mainly among peers: for example, a group of victims shared the same experience and developed a common strategy to cope with SH: “*Maybe she was a little more direct and aggressive than me. She would say, ‘Look, professor, I’m not interested, I don’t want to hear you’. Then, when he continued, she would either stop responding or say, ‘I’m at work’. We tried to have the same response method […], we formed a group, called ‘together’, because we were all in the same situation, where we exchanged messages received from the professor*”.

In other cases, a strong relationship with colleagues led the victim to ask for their help in avoiding direct contact with the harasser or being alone in the office. These are practical strategies for containing a critical situation through concrete behavioral support from others, thus demonstrating that colleagues do not necessarily need to have such empathic skills as to provide deep understanding and emotional support. Certainly, asking someone to adopt and consistently maintain certain behaviors means that there is a great deal of trust and confidence in the relationship with that colleague: it is a matter of making a commitment to support the coping strategies of the victim, whose personal narrative is therefore understood and shared.

“*I regularly put all my colleagues in CC when I received emails from him, so they could see his first email. In some cases, we had agreed that I would not pass on materials to him, but he (the colleague) would do so physically, I would just reply to him by email. This type of agreement aimed at limiting the discomfort, or rather the damage, in short, the annoyance of working with this person*”.

“*I decided to always have someone else present during meetings, to avoid being alone with him in the office. I chose a trusted colleague who had helped me with my work, as he had created a safe environment where I felt comfortable opening up. He was supportive […], often asking if I needed anything or if he should accompany me. Together, we took steps to ensure I was never alone in those situations with him*”.

#### 3.3.2. The Behavioral Extra-Fatigue: When Escape Is the Only Way out

As anticipated, the decision to report also often matures in a relational context, moved by the hope of “resolving” the issue, finding institutional validation of one’s experience and being able to contribute in sanctioning inappropriate behavior. This decision, described by one interviewee as “*the beginning of a nightmare*”, often leads to severe disappointment because of additional barriers challenging the victim’s ability to assert her “story” without suffering further backlash.

Faced with the huge disappointment of the system’s inability to adequately accommodate their experiences, the last possible protective strategy for victims is to leave the university. Once again, however, this requires extra effort because, for example, winning a scholarship abroad requires concentration and clear objectives, which conflict with the state of exhaustion of the victims, whose stories frequently mention the feeling of being “*in a state of confusion*”. This effort is well expressed in the following excerpt that illustrates the behavioral dimension of dialogical conflict: two incompatible narratives—the victim processing her trauma and the professional self required to perform competence—coexist within the same period, pulling in opposite directions. The exhaustion described is not merely physical but dialogical, produced by the impossibility of integrating these two voices: “*I remember […] the worst days of my life. I couldn’t sleep at night because I had thoughts: I thought about this thing, how to react, I was processing the harassment. I wondered, ‘How is it possible that I didn’t notice?’. I spent nights where I didn’t sleep a wink, but then I was awake during the day too because I was writing this application. […] It was agony, I had a terrible month […]. People who saw me again last year, after a long time… a colleague of mine who saw me during that month and then saw me again last year, when I was already abroad, said to me ‘You’re reborn, you’re a different person, I didn’t recognize you’”*.

In cases of students, after a formal reporting, the academic environment can become very difficult to bear, to the point that “*If I could go back, I wouldn’t do it again*”. For example, being forced to continue to have contact with people who are aware of the SH incident, can cause such discomfort that escape appears the inevitable path: “*The relationship with this professor in class, that I was forced to have, who was his friend […], it was awful, because I felt very fixated […] so much that I left, because I didn’t feel comfortable there*”. Moreover, perceiving institutional help and responses as ineffective presents one of the most common barriers to formal reporting: the fear of retaliation, which is intertwined with the asymmetrical dynamics of the academic context. Remaining at *that* university would mean continuing to deal with *that* professor, who, as such, could exploit his superior position to “make pay” for the complaint by, for example, giving unfair grades. This is how this anticipatory fear makes fleeing elsewhere seem like the best option.

“*I would have changed anyway because the course I wanted to study wasn’t available there […] but I cannot deny that if I had known that the professor would still have been teaching (after the formal reporting), I would probably have had some difficulty staying there*”.

However, escape does not always involve a change of physical environment, not least because this can sometimes cause financial damage due to the loss of a job. Therefore, escape can take the form of an emotional disengagement from the academic work environment, which allows the individual to remain physically present, while being absent in terms of motivation: “*Certainly, I had a consequent distrust of that structure, even in my relationships with some colleagues; I apparently continued to be a polite and cooperative person, professional, but I increasingly withdrew my investment in what I was doing and in that work situation*”.

## 4. Discussion

Through a narrative approach, this study investigated how victims of SH in academia experience such situations and what resources they activate when deciding to break the silence. By focusing on both informal disclosure to close peers, colleagues, or trusted professors, and to formal reporting within university, it was possible to reconstruct the red thread between individual, relational, and contextual levels [53,54] of academic socio-ecology. Personal narratives take shape through interaction with the academic environment, whose norms define how situations can be addressed. Our study confirmed that SH experiences generate a feeling of ambiguity that makes it difficult to be sure of what is happening, clearly highlighting «the injustice of having some significant area of one’s social experience obscured from collective understanding owing to a structural identity prejudice in the collective hermeneutic resource» [61] (p. 155). For this reason, many SH victims often remain silent and decide not to report, fearing that they will not be believed or not knowing whether the experience is worth reporting [59]. This is an extremely critical point because, as Avery, Schaubroeck & Sterckx [25] pointed out, «the possibility to interpret one’s own experiences and the possibility to talk about one’s experiences in a trusting environment are interdependent. The impact of an audience’s response on a survivor’s capacity to interpret their own experience is of huge importance». If faced with an audience that is unable to understand, or is even hostile, there is a risk that the victim herself will minimize or silence her own experience, which Dotson [89] defined as «testimonial smothering». Therefore, as clearly emerged from our interviews, external validation of personal perception becomes essential to begin coping with it; otherwise, a new confusing and exhausting path may begin.

At a cognitive level, recognizing SH is not an immediate act, but a gradual, often painful process shaped through exchanges with others. Informal conversations with trusted colleagues or formal consultations with institutional figures can provide external validation that helps victims frame their experience as legitimate harm rather than personal misunderstanding or overreaction. Frank [90] calls this process «narrative ambush»: it happens when stories that have no place in people’s «inner library» teach those people who they can be and what they can do. Conversely, when the social or institutional environment is ambivalent or downplaying, victims often internalize these interpretations, resulting in self-doubt, isolation, and narrative dissonance, that is, all experiences resulting from hermeneutical injustice [60,61]. Victims may turn to intellectualization to respond to this lack of validation: for example, engaging feminist and sociological literature may allow them to contextualize their experiences within cultural and structural frameworks, thus regaining narrative coherence. In line with previous studies that found support from friends or colleagues as a frequently effective coping strategy [23,45,51], another valuable tool is peer comparison, when shared experiences allow confirmation of the reality of personal SH incidents and overcoming feelings of isolation and self-blame. However, when some victims find the strength to formally report, they may face systemic inertia or inadequate institutional responses [91], thus recalling what Hershcovis and colleagues [92] defined as «network silence». The consequence for the victims is the perceived institutional betrayal [93] that must be explained and made sense of, reframing, intellectualizing, repeatedly re-narrating one’s account across interlocutors who may or may not validate it. This cognitive work constitutes the first layer of extra-fatigue.

The ability of victims to process SH is greatly supported by openness and sensitive support from others [43,45,51]. In the stories of our interviewees, empathetic bystanders, whether superiors or colleagues, became a safe haven offering understanding and dignity in situations of vulnerability. This emotional co-management reveals the softer and protective side of the academic world, helping victims regain their self-confidence and counteracting the shame resulting from hostile narratives. Conversely, formal institutional responses can add emotional stress, making victims feel exposed and ignored if they encounter coldness or minimization, as already discussed in depth by Smith & Freyd [94]. Such reactions may cause self-doubt and perceived isolation, above all when institutions treat disclosures as mere bureaucratic procedures [95]. However, over time, emotional distress can shift into anger, for example, when victims recognize the same patterns or connect with others who’ve experienced similar harm. This emotional change is often supported by feminist readings or peer solidarity, which foster deep awareness of the structural nature of SH. Absorbing institutional coldness, managing recurring self-doubt, and gradually transforming distress into collective awareness: this emotional labor constitutes the second layer of extra-fatigue, one that informal networks partially absorb but cannot eliminate.

Also external coping responses reveal the central role of relational dynamics in enabling protective actions and fostering resilience. Victims develop practical strategies, like coordinated responses or avoidance tactics [23,43], through trust-based relationships. While support from senior academics can empower without overriding agency, collective peer action most effectively sustains problem-focused coping. However, when formal reporting is perceived as ineffective, victims may resort to more drastic strategies like escape, by changing institutions, or emotionally disengaging from their work. Applying for a grant abroad, reorganizing one’s entire professional trajectory, learning to never be alone in a room with a perpetrator: these behavioral responses, often sustained only by the encouragement of trusted individuals in the absence of adequate institutional protection, constitute the third layer of extra-fatigue. Together, the three layers reveal that coping with SH is not only shaped by the victim’s capacity for action but is deeply embedded in the quality of the relational environment. Peers and informal ties appear to be the main relational resources capable of transforming a fragmented life story into a more coherent and meaningful whole [96].

Ahmed [73] (p. 5) observes that the story of a complaint is often a story of exhaustion: «exhausting, especially given that what you complain about is already exhausting». Building on this insight, and extending it beyond the formal complaint process to encompass the full arc of sense-making and help-seeking, we have introduced the concept of *extra-fatigue*: it is defined here as the cumulative cognitive, emotional, and practical labor that victims of SH are compelled to perform as a direct consequence of inadequate institutional responses. Unlike the harm caused by harassment itself, extra-fatigue is generated by the help-seeking trajectory: by the work of making sense of ambiguous experiences, building coalitions of informal support, repeatedly re-narrating one’s account across multiple interlocutors, and navigating institutional procedures that are structurally oriented toward self-protection rather than victim safety.

As a concept, extra-fatigue is distinct from, yet related to, several adjacent constructs in the literature. It differs from «secondary victimization» e.g., ref. [97], which refers to harm arising from others’ negative or dismissive reactions to a disclosure, in that extra-fatigue captures not a single harmful response but the cumulative labor generated across the entire help-seeking trajectory. It differs from «institutional betrayal trauma» [93,94], which emphasizes the psychological injury of betrayal by a trusted institution, in that extra-fatigue foregrounds the active, effortful work victims must perform—intellectualizing, re-narrating, coalition-building, escaping—precisely because that betrayal has occurred. It is closely related to what Ahmed [73] terms «complaint labor»: the largely invisible, uncompensated work of navigating institutional complaint procedures that are structurally oriented toward protecting the institution’s reputation rather than the complainant’s safety. Extra-fatigue extends this concept beyond formal complaints to encompass the full arc of sense-making and coping labor that precedes, surrounds, and follows any attempt at disclosure. Crucially, extra-fatigue should not be understood as an individual psychological burden to be mitigated through resilience-building or therapeutic support alone. Rather, it is structurally produced, being the cost that victims pay when institutions fail to fulfill their protective function and displace that labor onto victims and their informal supporters. As the three dimensions above illustrate, this cost is compounded for those already subject to minority stress—that is, the chronic stress produced by stigma and structural discrimination that falls on members of marginalized groups above and beyond general stressors [98]—for whom inadequate institutional responses do not merely add burden but deepen pre-existing marginalization. Understanding extra-fatigue as structurally offloaded institutional labor, rather than as a symptom of individual fragility, is essential for designing responses that address its root causes rather than merely its effects.

A key insight is the distinction between informal and formal interactions in accompanying the process of search and awareness initiated by the victim, supporting or not her narrative. The informal context may be traversed by contradictory narratives, in which, however, some people stand out for their ability to correctly frame the harassment victim’s experience, offer the space to take a breath, listen to a cry, or devise a strategy to never leave the victim alone. All these resources are part of the academic relational context and are the *humus* for preventive interventions. Therefore, connectedness [38] was confirmed as a protective factor, as well as the importance of working with a social-ecological perspective, strengthening relationships and a sense of community [6,94,99]. This underscores the necessity to surmount the perception that SH is rooted in individual behavior rather than recognizing its structural and systemic dimensions: socio-cultural aspects are both at its root and the key to countering it effectively.

Furthermore, our findings showed the strategic role of professors in supporting victims to cope with the situation, minimizing the harm. This valuable support confirms the importance of taking a perspective that involves the entire academic community, not only in tertiary prevention, as in this case, but also in the most important challenge of primary prevention [100,101]. A desirable approach in this direction is bystander intervention, which aims at empowering everyone in preventing and combating SH by providing the necessary tools [6,102]. The climate and culture of an environment, in which inappropriate behaviors manage to take shape, are the result of the prevailing thinking and actions of individuals; therefore, to create a safe space in which certain behaviors are no longer normalized, a cultural change is needed and it starts with changing the way individuals think, interact and behave [103].

When the informal network is supportive, the victim’s narrative is “solidified” and may come to the attention of institutional services or, if they do not exist or are not known, to people in positions of institutional authority. However, our research revealed that often, instead of acting as a tool for empowerment, formal reporting catapults victims into the ambiguity from which the narrative arose and to which it constantly tends to return. This happens because sometimes belittling narratives are encountered, or there is a lack of clarity about the path taken and its possible outcomes, which, in the eyes of the interviewees, were always vague and disappointing. Stauffer [104] examines this idea through her notion of «ethical loneliness», emphasizing the painful paradox that occurs when institutions meant to provide a voice instead refuse to truly hear. This dynamic reflects what Ahmed [105] (p. 17) identifies as a structural paradox at the heart of institutional complaint: «the reasons making a complaint is difficult are the same reasons that making a complaint is necessary». Complaint procedures are designed to address abuses of power, yet their operation often reproduces the very conditions—hierarchy, reputation management, strategic inefficiency—that produced the abuse. In this sense, the institutional response to harassment is itself a form of institutional harassment [105].

In this regard, several criticisms have been raised about «ornamental» interventions that align with the values of the neoliberal university, where institutional responses are shaped by economic and reputational considerations [106,107]. Performance and reputation are prioritized, often at the expense of addressing SH, leading to normalization of toxic behaviors to protect institutional image, rather than supporting victims, who, on the contrary, are often silenced [108,109]. This aspect particularly stands out when considering the mismatch within the same context, namely the university, between informal help from trusted people and formal help from the institution. This mismatch is particularly acute in the Italian academic context, where the “baronial” hierarchical system, in which senior professors exercise wide-ranging influence over the careers, funding, and mobility of junior colleagues, concentrates both the conditions producing SH and the authority to respond to it in the same institutional figures. When the perpetrator is a supervisor or department authority, formal reporting channels are not merely bureaucratically inadequate: they are structurally compromised, because the individuals best positioned to act are often those with the greatest reputational or relational stake in managing the incident quietly. This dynamic helps explain why formal responses in our data were experienced not simply as cold, but as actively reorienting victims away from complaint and toward accommodation.

In light of this, the most important challenge is finding a way to bridge this gap and align the informal level with the institutional one. The creation of formalized, but equally safe, networks in universities would reproduce the same *humus* that, as our results showed, is already being cultivated by a few trusted professors or close colleagues. Then, this bottom-up social fabric must be constructively engaged in dialogue with the higher institutional level towards a common goal: well-being and safety within academia [110].

## 5. Limitations

Several limitations of this study should be acknowledged. First, while the adequacy of the sample size has been discussed in relation to the epistemological premises of narrative inquiry, it is important to note that the accounts collected reflect the perspectives of those who were able and willing to speak—primarily individuals who had already achieved some degree of narrative coherence around their experience. As such, findings illuminate the dynamics of reporting and coping among those who broke the silence, rather than the full spectrum of SH experiences in Italian academia.

Second, the study captures victims’ narratives of how events were experienced and interpreted, leaving the institutional perspective unexplored; whether formal reporting led to concrete outcomes or procedural changes within universities remains outside the scope of this analysis. It is therefore not possible to determine from these accounts alone whether the failures described reflect individual competence gaps, institutional culture of reputation protection, or the binding constraints of Italian law—for example, the evidentiary requirements that the Confidential Counselor cited in one narrative as grounds for inaction. Distinguishing person-specific errors from structural legal limitations is an important direction for future research, which should triangulate victims’ accounts with institutional data, procedural documentation, and staff perspectives to understand where, precisely, the reporting process breaks down and what type of intervention (training, legal reform, structural redesign) is most likely to address it.

In addition, the sample size reduction from 12 to 8 interviews must be addressed. The four participants who did not report were not included in the thematic coding, given the study’s explicit focus on the reporting process and its relational dynamics. However, their accounts were read and discussed by both researchers as analytic counterpoints. These narratives were marked by an acute sense of isolation, a stronger perception that the incident would not be believed or minimized, and the students’ position—in three of four cases—that made any form of disclosure feel unsafe. While a full analysis of non-reporting narratives falls outside the scope of this paper, these accounts are consistent with the structural silencing dynamics identified in the reporting sample and suggest that the threshold for disclosure is shaped less by individual characteristics than by the perceived safety of the relational and institutional environment. We encourage future research to analyze non-reporting accounts at equivalent depth, as they are likely to illuminate the conditions that prevent the reporting process from beginning at all.

Finally, the lack of homogeneity of the sample should be noted. This certainly represents a limitation connecting and reiterating a gap repeatedly discussed in literature, namely the lack of an intersectional lens in research, with a tendency to address the “ideal survivor” (white, cisgender women) and overlook risks faced by marginalized groups, reinforcing stereotypes and exclusion. In our study, although it was open to everyone and not only to people with specific socio-demographic characteristics, those who took action and responded to the call were all (except one) cisgender women. This suggests that it is more difficult to reach marginalized groups, probably because they are more reluctant to expose themselves, perceiving that they would not be protected. Therefore, even though this is no easy challenge, this leaves room to investigate and explore these crucial aspects, thus fortifying a ground that studies like this are already making fertile for a real whole campus approach with a transformative impact [106].

## 6. Conclusions

Valuable implications for policy and future interventions emerge from this research, particularly about how institutions can improve responses and prevention of SH in academia. First, the discrepancy between supportive informal relationships and ineffective or emotionally detached formal procedures sheds light on the need for greater coherence and transparency in institutional narratives. Victims must feel heard and be clearly informed about the reporting process and its possible outcomes. Therefore, formal support mechanisms should be enhanced through trauma-informed practices that prioritize empathy and procedural fairness [20,25,111,112], avoiding the emotional disorientation identified across our narratives. At the same time, it is crucial to formally acknowledge the key role played by informal actors, such as trusted peers, colleagues, and supportive professors, by equipping them with the tools to offer initial support and to act as relational bridges toward formal services. Therefore, a hybrid model of care valuing both formal structures and informal networks could ensure that the supportive *humus* already present in certain academic relationships becomes an institutional resource rather than an isolated exception. We recognize, however, that formalizing informal support without accompanying structural reform risks institutionalizing the very displacement of care labor onto victims and their networks that this work has critiqued. For hybrid models to avoid reproducing this dynamic, informal supporters must be explicitly resourced—through designated time, basic training, institutional recognition, and meaningful protection from retaliation—rather than simply enlisted as unpaid bridges to procedures that remain inadequate. The following interconnected steps could put this into practice. First, universities should invest in peer awareness programs that go beyond traditional bystander intervention, equipping trusted colleagues, peers, and supportive professors not only to recognize and respond to harassment in the moment but to accompany victims through the help-seeking process: providing information about available services, normalizing the act of seeking support, and offering to be physically or emotionally present during initial contacts with formal structures. Second, Confidential Counselors and equivalent formal actors should be trained to receive disclosures that arrive through informal channels—that is, to work with victims who come accompanied by a peer or colleague and to treat that relational context as a resource rather than an obstacle to procedure. Third, universities should establish clear referral pathways that formalize the bridge between informal and formal support without eliminating the relational dimension: for example, a designated first-contact role that combines accessibility, confidentiality, and procedural knowledge and that can be activated by informal supporters on behalf of or alongside a victim. Overall, these steps would reduce the burden of navigation that currently falls entirely on the victim and her informal network.

The relational and procedural recommendations above are necessary but not sufficient. Our findings point to structural conditions that no amount of trauma-informed training or bystander programming can address on their own: the hierarchical dependency of students and early-career academics on individual supervisors within the Italian competitive examination system; the silencing function of temporary and precarious contracts, which make formal complaint disproportionately costly for those with the least institutional power; and the non-binding, advisory status of CUGs mechanisms, which limits their capacity to enforce meaningful accountability. Addressing these conditions requires structural interventions, including at minimum: ensuring that complaint mechanisms—whether Confidential Counselors, CUG bodies, or equivalents—are structurally independent from institutional reputation management, with guaranteed access for all contract types, including temporary and doctoral positions; introducing accountability mechanisms for senior faculty and administrators who minimize, dismiss, or fail to act on disclosures; and exploring funder-level levers, such as Horizon Europe and MUR grant conditions, that make verifiable institutional compliance a prerequisite for continued funding. Equally, the data from this study suggest that collective and union-based complaint routes warrant far greater attention: peer collective action—forming groups, sharing evidence, reporting jointly—emerged as one of the most effective behavioral strategies in our narratives, yet institutional frameworks rarely facilitate or protect it. Ahmed’s [73] analysis of complaint collectives corroborates this finding: collective complaint is not merely a tactical choice but a structural necessity when institutional channels are designed in ways that individualize risk and isolate complainers. Facilitating and protecting collective routes—including union-based mechanisms—is therefore not peripheral to institutional reform but central to it.

In this context, victims’ narratives should be considered powerful epistemic and moral resources. As Avery, Schaubroeck & Sterckx [25] brilliantly argued, storytelling is a means of «re-storying» the academic institution: not only to «do justice» but also to «undo injustice», recognizing the moral, social, and epistemic damage suffered. Creating archives of stories, spaces for listening, and policies informed by survivors’ narratives is a concrete step toward hermeneutical and moral justice in universities.

Moreover, culturally grounded interventions, like bystander programs tailored to the specific hierarchical dynamics of Italian academia, remain important complements to structural reform [110]. Institutions should also implement regular climate assessments and confidential feedback mechanisms for survivors to monitor the effectiveness of their interventions and build accountability and trust over time [20].

By integrating procedural, relational, and structural actions within a socio-ecological framework, academic institutions can move beyond ornamental responses and begin to address the systemic roots of SH. This requires, above all, a shift in institutional logic: from managing extra-fatigue as an individual burden to eliminating the structural conditions that produce it.

## Figures and Tables

**Table 1:** Characteristics of sexual harassment (SH) victims (and their perpetrators) interviewed.

Victim’s Academic Position	Victim Gender	*N* Interviewees	Power Relationship	Perpetrator Gender	*N* Eligible (With Report)
Students	Female	5	Subordinate	Male	2
Academic staff (temporary)	Female	4	Subordinate	Male	3
Academic staff (structured)	Female	1	Subordinate	Male	1
Administrative staff	Female	1	Equal	Male	1
Professor	Male	1	Contrapower	Female	1
Total	—	12	—	—	8

## Data Availability

The datasets generated and analyzed during the current study are not publicly available due to ethical restrictions and participant privacy protection but are available from the corresponding author upon reasonable request and with permission from the Human Research Ethics Committee.

## Supplementary Materials

The following supporting information can be downloaded at https://www.mdpi.com/article/10.3390/ijerph23050634/s1, Table S1: Analytical Data Coding Scheme.

## References

[1] United Nation (2008). Prohibition of Discrimination, Harassment, Including Sexual Harassment, and Abuse of Authority. Secretary-General’s Bulletin.

[2] Bondestam F., Lundqvist M. (2020). Sexual harassment in higher education—A systematic review. Eur. J. High. Educ..

[3] Benya F.F., Widnall S.E., Johnson P.A., National Academies of Sciences, Engineering, and Medicine (NASEM) (2018). Sexual Harassment of Women: Climate, Culture, and Consequences in Academic Sciences, Engineering, and Medicine.

[4] Eriksen S., Chib S.S., Katz J., Cortez-Barba Y., Rayburn P., Aldridge L. (2022). The best of times, the worst of times: Best practices for survivor support and gender violence prevention education on college campuses. Women Ther..

[5] Nowrouzi-Kia B., Chan H.Y., Zhu S., Nandan S., Bani-Fatemi A., Howe A., Gross D.P., Gohar B., Yazdani A., Chattu V.K. (2025). Examining the prevalence and effects of gender-based violence in academic settings: A systematic review and meta-analyses. Trauma Violence Abus..

[6] Sotirovic P., Blažytė G., Limantė A., Tereškinas A., Vaičiūnienė R. (2024). What makes academia (un)safe: Experiences, observations, and consequences of gender-based violence in different stages of individual researchers’ careers. Gender-Based Violence and the Law.

[7] Zara G., Binik O., Ginocchio D., Merzagora I., Giannini A., Addabbo T., Castelli L., Criscenti C., Ferrari S., Di Tella M. (2025). Looking for a preventive approach to sexual harassment in academia. A systematic review. Eur. J. Crim. Policy Res..

[8] Jones C., Chappell A., Alldred P. (2021). Feminist education for university staff responding to disclosures of sexual violence: A critique of the dominant model of staff development. Gend. Educ..

[9] Merzagora I., Giannini A., Zara G., Vesentini M., De Fazio G.L. (2022). #Wetoo? Sexual harassment in academia: Promoting a gender criminology. Rass. Ital. Criminol..

[10] Strid S., Humbert A.L., Hearn J., Bondestam F., Husu L. (2021). UniSAFE Ending Gender-Based Violence: Theoretical and Conceptual Framework. https://unisafe-gbv.eu/wp-content/uploads/2021/05/DT31_Theoretical-and-conceptual-framework_290421.pdf.

[11] USV React (2019). Training to Respond to Sexual Violence at European Universities: Final Report of the USVreact Project. http://usvreact.eu/it/about/.

[12] Zavos A. (2018). Sexual violence in Greek universities: Politics of disclosure, interventions and institutional change. Annu. Rev. Crit. Psychol..

[13] Boyle K.M., McKinzie A.E. (2021). The prevalence and psychological cost of interpersonal violence in graduate and law school. J. Interpers. Violence.

[14] Dion J., Boisvert S., Paquette G., Bergeron M., Hébert M., Daigneault I. (2022). Sexual violence at university: Are indigenous students more at risk?. J. Interpers. Violence.

[15] Hervías Parejo V. (2023). Sexual and sexist violence in the universities of southern Spain. J. Interpers. Violence.

[16] Vargas E.A., Brassel S.T., Perumalswami C.R., Johnson T.R., Jagsi R., Cortina L.M., Settles I.H. (2021). Incidence and group comparisons of harassment based on gender, LGBTQ+ identity, and race at an academic medical center. J. Women’s Health.

[17] Aguilar S.J., Baek C. (2020). Sexual harassment in academe is underreported, especially by students in the life and physical sciences. PLoS ONE.

[18] Bloom B.E., Sorin C.R., Wagman J.A., Oaks L. (2021). Employees, advisees, and emerging scholars: A qualitative analysis of graduate students’ roles and experiences of sexual violence and sexual harassment on college campuses. Sex. Cult..

[19] Kotze J.L., Frazier P.A., Huber K.A., Marcoulides K.M., Lust K.A. (2022). Identifying correlates of peer and faculty/staff sexual harassment in US students. J. Sex. Res..

[20] Crusto C.A., Hooper L.M., Arora I.S. (2024). Preventing sexual harassment in higher education: A framework for prevention science program development. J. Prev..

[21] O’Connor J., Smith L., Woerner J., Khan A. (2024). Protective factors for sexual violence perpetration among high school and college students: A systematic review. Trauma Violence Abus..

[22] Wood L., Hoefer S., Kammer-Kerwick M., Parra-Cordona J.R., Busch-Armendariz N. (2021). Sexual harassment at institutions of higher education: Prevalence, risk, and extent. J. Interpers. Violence.

[23] Cortina L.M., Wasti S.A. (2005). Profiles in coping: Responses to sexual harassment across persons, organizations, and cultures. J. Appl. Psychol..

[24] Wasti S.A., Cortina L.M. (2002). Coping in context: Sociocultural determinants of responses to sexual harassment. J. Personal. Soc. Psychol..

[25] Avery S., Schaubroeck K., Sterckx S. (2025). Re-storying sexual harassment in academia: The power of storytelling in combating epistemic injustice. Eur. J. Women’s Stud..

[26] Ahrens C.E. (2006). Being silenced: The impact of negative social reactions on the disclosure of rape. Am. J. Community Psychol..

[27] Starzynski L.L., Ullman S.E., Vasquez A.L. (2017). Sexual assault survivors’ experiences with mental health professionals: A qualitative study. Women Ther..

[28] Hall J.E., Simon T.R., Mercy J.A., Loeber R., Farrington D.P., Lee R.D. (2012). Centers for Disease Control and Prevention’s expert panel on protective factors for youth violence perpetration: Background and overview. Am. J. Prev. Med..

[29] Jolliffe D., Farrington D.P., Loeber R., Pardini D. (2016). Protective factors for violence: Results from the Pittsburgh Youth Study. J. Crim. Justice.

[30] Hollander J.A. (2018). Women’s self-defense and sexual assault resistance: The state of the field. Sociol. Compass.

[31] Kelley E.L., Gidycz C.A. (2017). Mediators of the relationship between sexual assault and sexual functioning difficulties among college women. Psychol. Violence.

[32] Senn C.Y., Eliasziw M., Hobden K.L., Newby-Clark I.R., Barata P.C., Radtke H.L., Thurston W.E. (2017). Secondary and 2-year outcomes of a sexual assault resistance program for university women. Psychol. Women Q..

[33] Fisher B.S., Daigle L.E., Cullen F.T., Santana S.A. (2007). Assessing the efficacy of the protective action–completion nexus for sexual victimizations. Violence Vict..

[34] Senn C.Y., Eliasziw M., Barata P.C., Thurston W.E., Newby-Clark I.R., Radtke H.L., Hobden K.L. (2015). Efficacy of a sexual assault resistance program for university women. N. Engl. J. Med..

[35] Turchik J.A., Probst D.R., Chau M., Nigoff A., Gidycz C.A. (2007). Factors predicting the type of tactics used to resist sexual assault: A prospective study of college women. J. Consult. Clin. Psychol..

[36] Decker M.R., Wood S.N., Ndinda E., Yenokyan G., Sinclair J., Maksud N., Ross B., Omondi B., Ndirangu M. (2018). Sexual violence among adolescent girls and young women in Malawi: A cluster-randomized controlled implementation trial of empowerment self-defense training. BMC Public Health.

[37] Edwards K.M., Siller L., Leader Charge D., Bordeaux S., Leader Charge L. (2022). Dating violence, sexual assault, and sexual harassment victimization among girls on an Indian reservation: An examination of rates and risk and protective factors. Violence Against Women.

[38] Perez-Trujillo M., Jaramillo-Sierra A.L., Quintane E. (2019). Connectedness as a protective factor of sexual victimization among university students. Vict. Offenders.

[39] Bell M.P., Quick J.C., Cycyota C.S. (2002). Assessment and prevention of sexual harassment of employees: An applied guide to creating healthy organizations. Int. J. Sel. Assess..

[40] Mujal G.N., Taylor M.E., Fry J.L., Gochez-Kerr T.H., Weaver N.L. (2021). A systematic review of bystander interventions for the prevention of sexual violence. Trauma Violence Abus..

[41] Fitzgerald L.F., Swan S., Fischer K. (1995). Why didn’t she just report him? The psychological and legal implications of women’s responses to sexual harassment. J. Soc. Issues.

[42] Gruber J.E. (1989). How women handle sexual harassment: A literature review. Soc. Sci. Res..

[43] Fitzgerald L.F., Cortina L.M., Travis C.B., White J.W., Rutherford A.E., Williams W.S., Cook S.L., Wyche K.F.E. (2018). Sexual harassment in work organizations: A view from the 21st century. APA Handbook of the Psychology of Women: Perspectives on Women’s Private and Public Lives.

[44] Magley V.J. (2002). Coping with sexual harassment: Reconceptualizing women’s resistance. J. Personal. Soc. Psychol..

[45] Scarduzio J.A., Sheff S.E., Smith M. (2018). Coping and sexual harassment: How victims cope across multiple settings. Arch. Sex. Behav..

[46] Fitzgerald L.F. Assessing strategies for coping with harassment: A theoretical/empirical approach. Proceedings of the Midwinter Conference of the Association for Women in Psychology.

[47] Fitzgerald L.F., Shullman S.L., Bailey N., Richards M., Swecker J., Gold Y., Ormerod M., Weitzman L. (1988). The incidence and dimensions of sexual harassment in academia and the workplace. J. Vocat. Behav..

[48] Folkman S., Lazarus R.S. (1989). Manual for the Ways of Coping Inventory.

[49] Lazarus R.S., Folkman S. (1984). Stress, Appraisal, and Coping.

[50] Culbertson A.L., Rosenfeld P., Booth-Kewley S., Magnusson P. (1992). Assessment of Sexual Harassment in the Navy: Results of the 1989 Navy-Wide Survey (TR-92-11).

[51] Mirhosseini Z., Pakdel P., Ebrahimi M. (2023). Women, sexual harassment, and coping strategies: A descriptive analysis. Iran. Rehabil. J..

[52] Schneider B.E. (1991). Put up and shut up: Workplace sexual assaults. Gend. Soc..

[53] Bronfenbrenner U. (1977). Toward an experimental ecology of human development. Am. Psychol..

[54] Bronfenbrenner U. (2005). Making Human Beings Human: Bioecological Perspectives on Human Development.

[55] Wold B., Mittelmark M.B. (2018). Health-promotion research over three decades: The social-ecological model and challenges in implementation of interventions. Scand. J. Public Health.

[56] Tharp A.T., DeGue S., Valle L.A., Brookmeyer K.A., Massetti G.M., Matjasko J.L. (2013). A systematic qualitative review of risk and protective factors for sexual violence perpetration. Trauma Violence Abus..

[57] Robinson S.R., Elias-Lambert N., Casiano A., Ward L. (2020). “Culture-bearer, culture-sharer, culture-changer”: The role of faculty in preventing sexual violence on campus. Adv. Soc. Work.

[58] Cottone N. (2023). Addressing the “puzzle” of gray-area sexual violations. Hypatia.

[59] Lipinsky A., Schredl C., Baumann H., Humbert A., Tanwar J. (2022). Gender-Based Violence and Its Consequences in European Academia: First Results from the UniSAFE Survey. https://unisafe-gbv.eu/wp-content/uploads/2022/11/UniSAFE-survey_prevalence-results_2022.pdf.

[60] Falbo A. (2022). Hermeneutical injustice: Distortion and conceptual aptness. Hypatia.

[61] Fricker M. (2007). Epistemic Injustice: Power and the Ethics of Knowing.

[62] Hussain S.T. (2024). Challenging gaslighting of women in academia: A call for gender equality and inclusive environment. Int. J. Innov. Teach. Learn..

[63] Penttinen E. (2023). “Collective gaslighting” and emotional workplace abuse in feminist academic spaces. Int. Fem. J. Politics.

[64] Fernando D., Prasad A. (2019). Sex-based harassment and organizational silencing: How women are led to reluctant acquiescence in academia. Hum. Relat..

[65] Maercker A., Horn A.B. (2013). A socio-interpersonal perspective on PTSD: The case for environments and interpersonal processes. Clin. Psychol. Psychother..

[66] Mitra A., Swendeman D., Sumstine S., Sorin C.R., Bloom B.E., Wagman J.A. (2022). Structural barriers to accessing the campus assault resources and education (CARE) offices at the University of California campuses. J. Interpers. Violence.

[67] Zinzow H.M., Thompson M. (2011). Barriers to reporting sexual victimization: Prevalence and correlates among undergraduate women. J. Aggress. Maltreat. Trauma.

[68] Pinchevsky G.M., Magnuson A.B., Augustyn M.B., Rennison C.M. (2019). Sexual victimization and sexual harassment among college students: A comparative analysis. J. Fam. Violence.

[69] Vargas E.A., Cortina L.M., Settles I.H., Brassel S.T., Perumalswami C.R., Johnson T.R., Jagsi R. (2022). Formal reporting of identity-based harassment at an academic medical center: Incidence, barriers, and institutional responses. Acad. Med..

[70] Cortina L.M., Magley V.J. (2003). Raising voice, risking retaliation: Events following interpersonal mistreatment in the workplace. J. Occup. Health Psychol..

[71] Pijlman V., Eichelsheim V., Pemberton A., Waardt M.D. (2025). “I did not want to make a bigger deal out of it than it was”: A mixed-method study on the help-seeking behavior of victims of image-based sexual harassment and abuse. J. Interpers. Violence.

[72] Kirkner A.C., Lorenz K., Mazar L. (2022). Faculty and staff reporting and disclosure of sexual harassment in higher education. Gend. Educ..

[73] Ahmed S. (2021). Complaint!.

[74] Gill R., Flood R., Gill R. (2009). Breaking the silence: The hidden injuries of neo-liberal academia. Secrecy and Silence in the Research Process: Feminist Reflections.

[75] Ivancheva M., Lynch K., Keating K. (2019). Precarity, gender and care in the neoliberal academy. Gend. Work Organ..

[76] Pemberton A., Aarten P.G., Mulder E. (2019). Stories as property: Narrative ownership as a key concept in victims’ experiences with criminal justice. Criminol. Crim. Justice.

[77] Walklate S., Maher J.M., McCulloch J., Fitz-Gibbon K., Beavis K. (2019). Victim stories and victim policy: Is there a case for a narrative victimology?. Crime Media Cult..

[78] Cook E.A., Walklate S. (2019). Excavating victim stories: Making sense of agency, suffering and redemption. Emerald Handbook of Narrative Criminology.

[79] Hourigan K.L. (2019). Narrative victimology: Speaker, audience, timing. Emerald Handbook of Narrative Criminology.

[80] Lindemann Nelson H. (2001). Damaged Identities, Narrative Repair.

[81] MacIntyre A.C. (1984). After Virtue: A Study in Moral Theory.

[82] Brison S.J. (2002). Aftermath: Violence and the Remaking of a Self.

[83] Vossler C., Presser L., Mulder E. (2024). “A spider on your shoulder”: Workplace sexual harassment through a narrative lens. Fem. Criminol..

[84] DeSouza E., Fansler A.G. (2003). Contrapower sexual harassment: A survey of students and faculty members. Sex Roles.

[85] Presser L. (2010). Collecting and analyzing the stories of offenders. J. Crim. Justice Educ..

[86] Sandberg S. (2022). Narrative analysis in criminology. J. Crim. Justice Educ..

[87] Braun V., Clarke V. (2021). Thematic Analysis: A Practical Guide.

[88] Hill C.E., Thompson B.J., Williams E.N. (1997). A gude to conducting consensual qualitative research. Couns. Psychol..

[89] Dotson K. (2011). Tracking epistemic violence, tracking practices of silencing. Hypatia.

[90] Frank A.W. (2010). Letting Stories Breathe: A Socio-Narratology.

[91] Täuber S. (2022). Women academics’ intersectional experiences of policy ineffectiveness in the European context. Front. Psychol..

[92] Hershcovis M.S., Vranjes I., Berdahl J.L., Cortina L.M. (2021). See no evil, hear no evil, speak no evil: Theorizing network silence around sexual harassment. J. Appl. Psychol..

[93] Smith C.P., Freyd J.J. (2014). Institutional betrayal. Am. Psychol..

[94] Smith C.P., Freyd J.J. (2013). Dangerous safe havens: Institutional betrayal exacerbates sexual trauma. J. Trauma. Stress.

[95] Hansen J., Nilsson I. (2022). Critical Storytelling: Experiences of Power Abuse in Academia.

[96] McAdams D.P., Josselson R., Lieblich A. (2006). Identity and Story: Creating Self in Narrative.

[97] Campbell R., Raja S. (1999). Secondary victimization of rape victims: Insights from mental health professionals who treat survivors of violence. Violence Vict..

[98] Meyer I.H. (2003). Prejudice, social stress, and mental health in lesbian, gay, and bisexual populations: Conceptual issues and research evidence. Psychol. Bull..

[99] Dahlberg L.L., Krug E.G., Krug E., Dahlberg L.L., Mercy J.A., Zwi A.B., Lozano R. (2002). Violence: A global public health problem. World Report on Violence and Health.

[100] Dills J., Fowler D., Payne G. (2016). Sexual Violence on Campus: Strategies for Prevention.

[101] DeGue S., Valle L.A., Holt M.K., Massetti G.M., Matjasko J.L., Tharp A.T. (2014). A systematic review of primary prevention strategies for sexual violence perpetration. Aggress. Violent Behav..

[102] Stojanov Z., Treharne G.J., Graham K., Liebergreen N., Shaw R., Hayward M., Beres M. (2021). Pro-social bystander sexual violence prevention workshops for first year university students: Perspectives of students and staff of residential colleges. Kōtuitui N. Z. J. Soc. Sci. Online.

[103] Beres M.A., Treharne G.J., Stojanov Z. (2019). A whole campus approach to sexual violence: The University of Otago model. J. High. Educ. Policy Manag..

[104] Stauffer J. (2015). Ethical Loneliness: The Injustices of Not Being Heard.

[105] Ahmed S. (2018). Complaint as feminist pedagogy. Annu. Rev. Crit. Psychol..

[106] Colpitts E.M. (2022). ‘Not even close to enough’: Sexual violence, intersectionality, and the neoliberal university. Gend. Educ..

[107] Gardiner R.A., Finn H. (2022). Implementing gender-based violence policies in the neoliberal university: Challenges and contradictions. Gend. Manag. Int. J..

[108] O’Connor P., Hodgins M., Woods D.R., Wallwaey E., Palmen R., Van Den Brink M., Schmidt E.K. (2021). Organisational characteristics that facilitate gender-based violence and harassment in higher education?. Adm. Sci..

[109] Phipps A. (2020). Reckoning up: Sexual harassment and violence in the neoliberal university. Gend. Educ..

[110] Ginocchio D., Binik O., Lausi G., Ferrari S., Addabbo T., Merzagora I., Giannini A., De Fazio G.L., Zara G. (2025). Sustainable Measures To End gender-based Violence in Academia: What Lies between the ERA Policy Paradigm and Reality?. Eur. J. Crim. Policy Res..

[111] Goldsmith R.E., Martin C.G., Smith C.P. (2014). Systemic trauma. J. Trauma Dissociation.

[112] McCauley H.L., Casler A.W. (2015). College sexual assault: A call for trauma-informed prevention. J. Adolesc. Health.

