# Supplementary material for: “Okay, So It’s Not Me”—The Extra-Fatigue of Formal and Informal Reporting of Sexual Harassment in Academia
Source: Int J Environ Res Public Health. 2026 May 11;23(5):634. doi: 10.3390/ijerph23050634 (PMC13206339; doi:10.3390/ijerph23050634)
Supplement: Supplementary file 1 [file ijerph-23-00634-s001.zip › ijerph-4280143-supplementary.pdf]

Table S1. Analytical Data Coding Scheme

Dimension / Subtheme	Code	Illustrative Excerpt (abbreviated)	Reporting Channel
**1. Cognitive Dimension**			
**1.1 Framing the situation through the eyes of others**	External validation of personal narrative	"What you experienced can be defined as SH"	Formal
	Peer mirroring / shared experience	"Okay, so it's not me"	Informal
	Narrative invalidation → self-doubt	"The context around me told me it was no big deal"	Informal
	Collective coalition building → formal report	"She and I decided to report it"	Informal → Formal
**1.2 Intellectualization as sense-making**	Feminist literature as cognitive reframe	"Exploring this topic from an intersectional feminist perspective opened my mind"	Informal
	Academic study as response to isolation	"I've started studying this topic: the literature agrees with me"	Formal
**1.3. The cognitive extra-fatigue: making sense of institutional inertia**	System failure → rationalization	"Internalized misogyny [...] many people lived in contexts that were still patriarchal, rigid [...] and internalized this vision"	Formal
**2. Emotional Dimension**			
**2.1 Openness and emotional support from the academic network**	Colleague as emotional safe haven	"No one will believe the word of someone like that"	Informal
	Bystander affirmation / anger as validation	"This is unacceptable"	Informal
	Decompression space	"I asked her if I could stay there for a while"	Informal
**2.2 The emotional extra-fatigue: dealing with distress and anger**	Institutional coldness → emotional isolation	"She was very cold, like a bureaucrat [...] it made me feel very alone"	Formal
	Anger as emotional turning point	"I was understandably angry: when it suited the university, my testimony was useful"	Formal
	System failure → emotional closeness	"We became close friends [...] what we all felt was helplessness"	Informal
	Institutional coldness → professional empathetic support	"She was the only one who was truly shocked, who made me talk"	Formal

Dimension / Subtheme	Code	Illustrative Excerpt (abbreviated)	Reporting Channel
**3. Behavioral Dimension**			
**3.1 Operational coping strategies**	Coordinated peer response strategy	"We tried to have the same response method [...] we formed a group called 'together'"	Informal
	Colleague as physical buffer	"I decided to always have someone else present during meetings"	Informal
	Email CC strategy / documentation	"I regularly put all my colleagues in CC when I received emails from him"	Informal
**3.2 The behavioral extra-fatigue: when escape is the only way out**	Escape via scholarship/mobility abroad	"It was agony [...] I was processing the harassment while writing the application"	Formal
	Physical exit from university	"I left, because I didn't feel comfortable there"	Formal
	Personal disengagement / staying physically	"I increasingly withdrew my investment in that work situation"	Formal

Note. Excerpts are abbreviated for readability. Full quotes appear in the Results section.
